# Stunting and its associated factors among children living with HIV/AIDS: a cross-sectional study

**DOI:** 10.1097/MS9.0000000000001961

**Published:** 2024-03-18

**Authors:** Zewdu Dagnew, Zeleke Mengist, Cheru Tesema, Tegegne Temesgen, Lake Kumlachew, Abraham Teym, Getasew Yirdaw, Yenewa Bewket, Zemene Addisie, Kemachew Ayele, Agernesh Ayele, Balew Adane, Eniyew Tegegne

**Affiliations:** Departments of a Environmental Health; b Public Health; c Human Nutrition, College of Health Sciences, Debre Markos University, Debre Markos, Ethiopia

**Keywords:** ART, Ethiopia, HIV/AIDS, nutrition, stunting

## Abstract

**Background:**

Growth failure is a common feature of children living with HIV/AIDS. This study was intended to assess the level of stunting and its associated factors among children living with HIV/AIDS.

**Methods:**

An institution-based cross-sectional study was conducted among 218 children living with HIV/AIDS. An interviewer-administered data collection tools and anthropometric measurements were used to collect data. Bivariable and multivariable logistic regression analyses were used to identify independent variables. Adjusted odds ratio with a 95% CI at a *P* value of 0.05, which was considered to declare statistical significance.

**Result:**

The level of stunting among children infected with HIV/AIDS in selected northwest comprehensive specialized hospitals in western Amhara was 56.9%. Poor anti-retroviral therapy (ART) adherence [adjusted odds ratio (AOR)=6.15, 95% CI, (3.88–9.69)], lack of co-trimoxazole prophylaxis [AOR=2.0, 95% CI, (1.88–2.98)], opportunistic infection [AOR=4.66, 95% CI, (3.24–6.11), and feeding twice [AOR=3.88, 95% CI, (1.94–5.14)] and feeding three times [AOR=1.52, 95% CI, (1.23–3.89)] were significantly associated with stunting.

**Conclusion:**

Stunting among HIV/AIDS-infected children was very high. Poor ART adherence, lack of co-trimoxazole prophylaxis, opportunistic infection, and low feeding frequency were significantly associated to stunting among HIV/AIDS-infected children. Strategies need to be devised to address factors amenable to modification to improve the growth of children living with HIV/AIDS.

## Introduction

Highlights

- The level of stunting among HIV-positive children was high.
- Hence, the high prevalence of stunting in children living with HIV/AIDS in this study reflects the need for an expanded effort to address it intensively.
- Poor anti-retroviral therapy (ART) adherence, lack of co-trimoxazole prophylaxis, opportunistic infection, and low feeding frequency were significantly associated with stunting among HIV/AIDS-infected children.
- Interventions need to be targeted to improve ART adherence, early prophylaxis, the treatment of opportunistic infections, and feeding frequency.

Childhood stunting is a condition that is defined as height for an age below the fifth percentile on a reference growth curve^1^. It is an important indicator of the prevalence of malnutrition or other nutrition-related disorders. Growth failure and metabolism abnormalities are common features of children living with the HIV. Undernutrition, especially stunting, increases mortality and may impair the response to anti-retroviral treatment^2^.

Globally, stunting is the major underlying cause of death in children under 5 years of age. Approximately 155 million (22.9%) under-five children suffer from stunting; of these, 38% live in Africa; particularly in Eastern Africa, which ranges about 36.7%^3^. More than six (6.3) million children under age 5 died in 2013, nearly 17 000 every day, and worldwide, about 10.9 million children less than five years old die each year, mostly due to undernutrition, especially stunting and HIV/AIDS^4^.

For children living with HIV, linear growth is usually the first parameter negatively affected by HIV disease progression and is prone to be exposed to different problems such as inadequate food intake, increased nutrient losses, and increased nutrient needs because of the hyper-metabolic and hyper-catabolic effects of infections, opportunistic infections, and HIV itself^5^. Infections and nutritional deficiencies cause an increase in pro-oxidants, resulting in oxidative stress, which may indirectly accelerate HIV replication^6^.

The mechanism for growth failure in HIV infection involves inadequate caloric intake, gastrointestinal infestations, opportunistic infections, abnormal resting energy expenditure, and endocrine abnormalities^7^. HIV-positive children need extra vitamins and minerals to help repair and heal damaged cells. They need to eat foods high in vitamins and minerals, which can help boost the immune system. Energy requirements are likely to increase by 10% to maintain growth in asymptomatic children^8^. In addition, poor growth in children living with HIV may have many causes, including reduced food intake due to socio-economic circumstances or, poor caregiving practices, such as when the mother is unwell, and opportunistic infections that can affect food intake, absorption, and metabolism^9^.

A number of causal factors suggest that the genesis of growth disturbances among children living with HIV is multi-factorial and multi-sectoral. Stunting is often caused by recognizable illnesses and secondary conditions that supplement HIV infection. Secondary causes of growth failure include dietary insufficiency, diarrhoeal diseases, and anaemia. Stunting is also encountered in children living with HIV children with no secondary illnesses. Stunting is also a result of multiple hard times, including food insecurity, poverty, and rampant disease, especially HIV/AIDS^3^.

Different stakeholders have taken different interventions to prevent childhood stunting globally and nationally. The interventions taken to tackle children’s stunting status are nutritional interventions (supplementation, micronutrient-fortified food or complementary food, promotion of nutrition), health interventions (reproductive and child health immunization, and increased access to health services with performance pay), WASH interventions (sanitation programs and community-based hand washing programs), and safety net programs (conditional cash transfer)^10^. In children living with HIV/AIDS, nutrition intervention and suppression of viral replication with ARVs are important means of tackling stunting^11^. However, despite the seriousness of the problem, studies conducted to explain factors affecting the stunting status of children living with HIV/AIDS are inadequate in general and in this study area in particular. Therefore, this study will fill the gap in the current situation by assessing the level of stunting and associated factors among children living with HIV/AIDS.

## Methods and material

### Study area, period, and design

An institution-based cross-sectional study was conducted from November to December 2019. One of the study areas is located in Debre Markos town, which is the capital of East Gojjam and is 299 km from Addis Ababa, the capital city. Whereas the second study area is a tertiary Comprehensive Specialized hospital with around 400 beds and nine operating tables that serves over 7 million people in the surrounding area. The area is 563 km from Addis Ababa, the country's capital city.

### Populations

#### Source population

All children living with HIV/AIDS who were under follow-up and treatment were the source population of this study.

#### Study population

Children under the age of fifteen living with HIV/AIDS who were under follow-up and treatment were the study population.

### Inclusion and exclusion criteria

#### Inclusion criteria

All caretakers (18 years and older) who came to the hospital for regular follow-up during the data collection period were included in the study.

#### Exclusion criteria

All caretakers who came to the hospital in the anti-retroviral therapy (ART) clinic for regular follow-up during the data collection period and could not communicate with the data collectors were excluded from the study.

### Sample size determination

The sample size was determined by using the single population proportion formula by taking prevalence from the study done on nutritional status among HIV-positive children in Harar, Eastern Ethiopia (49.1%)^12^ stunting in under-five children with a 95% CI and precision of 5% and a 10% non-response rate.


n=z²a/2*p*(1−p)/d²

*P*=0.49 assumed level of stunting


q=1−P=(1−0.49)=0.51

Z_*a*/2_= a standard normal value which corresponds 95% CI=1.96


n=(1.96)(1.96)(0.49)(0.51)/.05*.05

n=384

Since the study population (N) is less than 10 000, the final sample size was adjusted. *n*f=*n*/(1+*n/N*)=384/ (1+384/410)=198. After adding a 10% non-response rate, the final sample size was found to be 218. The sampling interval for the two comprehensive specialized hospitals was 2. That is for the one, *k*=122/65=1.8–2, and for the other, k=288/145=1.9–2.

### Sampling technique

**Figure 1:**
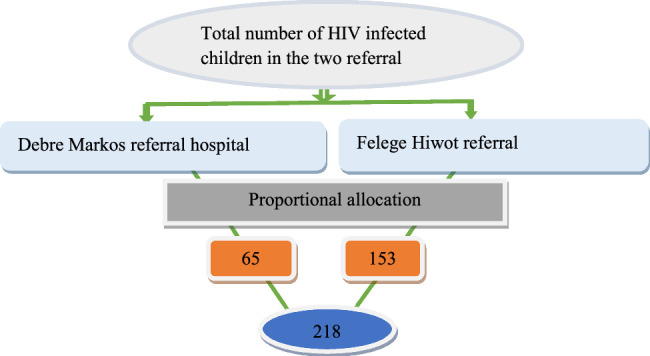
Schematic presentation of sampling procedure of HIV/AIDS-infected children who were on anti-retroviral therapy follow-up, 2019.

Under-fifteen children living with HIV who were on treatment and receiving regular follow-up in an ART clinic were selected from each Comprehensive Specialized hospital for an exit interview and documentary review. Patients or clients in their hospital were selected proportionally by systematic random sampling technique using a computer-registered list of children living with HIV in the Comprehensive Specialized hospitals in the ART clinic as a sampling frame. Study participants were selected using systematic sampling techniques from each comprehensive specialized hospital. The sample was allocated to the two hospitals proportionally. The first study participant was planned to be selected by lottery method from the two, and the rest were taken based on the interval. Finally, caretakers of the under-15 child living with HIV who fulfilled the inclusion criterion were interviewed by data collectors in each of the two comprehensive specialized hospitals (Fig. 1).

### Operational definitions

#### Stunting

when the anthropometric Z-score of the child falls −2SD below the median of the reference population^13^.

#### HIV infection

For those less than 18 months of age, a single HIV virology test of DNA PCR positive test result was taken as HIV infection, while for those age 18 months and above, HIV serologic (rapid HIV antibody) positive test result was taken as HIV infection.

#### Height

Length measurement for children below 24 months was taken in recumbent position, and standing height was taken for children 2–15 years, and the measurement was taken to the nearest 0.1 cm using Short’s Height Measuring Board (Short Productions), with the subjects shoeless].

#### Age

The child’s age was collected from the mother and confirmed by using birth certificates or vaccination cards, and we used a “local-events calendar.”

### Data collection and quality control procedures

Data were collected by interviewing eligible subjects using a pretested, semi-structured questionnaire for this purpose. The questionnaire was pretested among 5%(11) of the sample size. Its face validity, or if the test’s content seems appropriate for the goals that were thought to be included in it, and its content validity, or whether the test is completely representational of what it seeks to measure, were ensured^14^. The reliability was checked by Cronbach’s alpha (*P* value=0.89). Data were prepared for analysis by being cleaned, verified as complete, coded, and entered into SPSS 20. Five clinical nurses were used for data collection, and two bachelor nurses for supervision. Prior to the commencement of the actual data collection, one day of training was given to both data collectors and supervisors. Evening discussions were held during the time of data collection to identify the key findings, challenges, and other issues that the interviewers may have raised. The entire questionnaire was checked daily to see if it was filled out correctly.

### Data management and statistical analysis

The collected data were entered in Epidata version 3.1 and exported to SPSS version 20 for analysis. An anthropometric indicator, the height-for-age Z-score (HAZ), was calculated using ENA for SMART 2011 software (WHO Child Growth Standards 2006)^13^. The 2006 WHO reference standard was used to define stunting. Stunting was diagnosed when the anthropometric Z-score of the child fell −2SD below the median of the reference population. Frequencies, percentages, and summary statistics were computed to describe the study population in relation to relevant variables. Logistic regression (both bivariable and multivariable) was conducted to identify the factors that have been associated with stunting. A bivariable analysis was performed to see the association between dependent and independent variables. Variables with a *P* value less than or equal to 0.2 in the bivariable analysis were considered in the multivariable logistic regression analysis. Multivariable logistic regression analysis was employed to control for possible confounding effects and assess the separate effects of each variable. An adjusted odd ratio with a respected 95% confidence interval and a *P* value less than or equal to 0.05 was used as a cutoff point to declare statistically significant associations. The work has been reported in line with the STROCSS criteria^15^.

## Result

### Socio-demographic characteristics

A total of 210 study participants, 153 (70.18%) from Felege Hiwot and 65 (29.82%) from Debre Markos Comprehensive Specialized Hospitals, were included in this study, providing a response rate of 100%. This study had 115 (52.75% male) and 103 (47.25% female) children. The mean age of children included was 8.6 years, ranging from 1 to 14 years. Most children were aged 6–10 years, while 44 (20.18%) were under 5 years of age. Fifty (22.93%) of the caregivers were unable to read and write; 24 (11.0%) had attended primary school; and more than half of the caregivers, 144 (66.05%), had attended secondary school and college education. The median family income was 2700 birr per month. The family income ranged from 500 to 8000 birr per month. In addition, more than half (56.2%) were getting more than 1500 Birr per month. Nearly two-thirds (61.9%) of the caretakers were married. The feeding frequency of the children living with HIV in the past 24 h preceding the day of the data collection was 2–4 times per day, with 3 times per day being the median feeding frequency (Table 1).

**Table 1:** Socio-demographic characteristics of children living with HIV/AIDS who had follow-up, 2019 (*n*=218)

Variable	Category	Frequency	[%]
Age of the child in years	<3 years	3	1.4
	3–5 years	27	12.4
	6–10 years	122	56
	>10 years	66	30.3
Sex of the child	Male	115	52.8
	Female	103	47.2
Age of the mother of the child	19–24	28	12.8
	25–35	131	60.1
	36–49	59	27.1
Occupation of the mother of the child	Government	50	22.9
	Private	33	15.1
	Merchant	51	23.4
	Homemaker	29	13.3
	Farmer	29	13.3
	Daily labourer	26	11.9
Educational status	Not attended formal education	50	22.9
	Primary (1–8)	24	11
	Secondary (9–12)	80	36.7
	College	64	29.4
Family income	<750 birr	49	22.5
	751–1500 birr	36	16.5
	>1500 birr	133	61
Family size	<4	124	56.9
	4–6	65	29.8
	>6	29	13.3
Feeding frequency	Twice	52	23.85
	3 times	101	46.33
	4 times	65	29.82

### Clinical characteristics

All 218 children studied were on ART at the time of the survey. The median age of ART initiation for these children was 36 months. The median age to be diagnosed with HIV infection and the median age of starting follow-up in the hospitals were the same (16 months), ranging from 4 to 120 months. Fifty-one (23.4%) of the children were taking cotrimoxazol prophylactic therapy at the time of this study. One hundred thirty-four (61.47%) children reported good ART adherence. Of those children who were taking ART, only 31 (14.2%) had documented opportunistic infections during the study (Table 2).

**Table 2:** Clinical characteristics of children living with HIV, 2019 (*n*=218)

Variable	Category	Frequency	[%]
WHO clinical stage of HIV/AIDS	Stage I	131	60.03
	Stage II	23	10.61
	Stage III	33	15.1
	Stage IV	31	14.2
Is the child taking co-trimoxazole prophylactic	Yes	51	23.4
	No	167	76.6
Opportunistic infection	Yes	37	10.6
	No	312	89.4
ART adherence	Poor	84	38.53
	Good	134	61.47

ART, anti-retroviral therapy.

### Child stunting status

**Figure 2:**
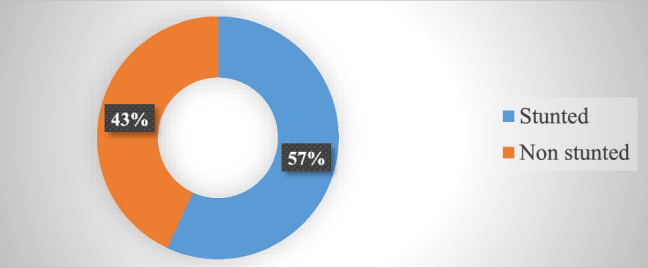
Proportion of stunting among HIV/AIDS-infected children who are on anti-retroviral therapy follow-up, 2019.

The level of stunting among children living with HIV/AIDS was found among 124 [56.90%, 95 CI, (49.4–58.6)] children (Fig. 2).

### Factors associated with stunting of children living with HIV/AIDS

Feeding frequency, mother’s occupation, family size, WHO clinical stage of a child living with HIV, mother’s level of education, opportunistic infection, monthly family income, adherence to ART, and co-trimoxazole prophylaxis were candidate variables for the multivariable logistic regression in the bivariable logistic regression analysis. In the multivariable logistic regression, poor ART adherence [adjusted odds ratio (AOR)=6.15, 95% CI, (3.88–9.69)], lack of co-trimoxazole prophylaxis [AOR=2.0, 95% CI, (1.88–2.98)], opportunistic infection [AOR=4.66, 95% CI, (3.24–6.11), and low feeding twice [AOR=3.88, 95% CI, (1.94–5.14)] and feeding three times [AOR=1.52, 95% CI, (1.23–3.89)] were significantly associated with stunting among children living with HIV/AIDS (Table 3).

**Table 3:** Bivariate and multivariable analysis showing factors associated with stunting among children living with HIV/AIDS, 2019.

	Stunting				
Variables	Yes	No	COR (95% CI)	AOR (95% CI)	*P*
Occupation of the mother					
Government employed	18	32	1	1	
Private employed	20	13	0.36 (0.24, 1.70)	0.32 (0.18, 1.5)	0.14
Merchant	28	23	2.16 (1.32, 3.78)	2.04 (1.37, 3.23)	0.41
Housewife	13	16	1.44 (0.80,1.89)	1.38 (0.49, 1.07)	0.284
Farmer	23	6	6.81 (3.81, 21.27)	4.13 (2.05, 17.20)	0.089
Daily labourer	22	4	9.77 (4.39, 11.39)	6.10 (3.394, 8.59)	0.438
Educational status of the mother					
Not attended	40	10	1	1	
Primary	13	11	0.140 (0.059, 0.332)	0.425 (0.095, 1.895)	0.262
Secondary	48	22	0.475 (0.183, 1.229)	0.878 (0.300, 2.575)	0.813
College	23	41	0.374 (0.190, 0.737)	0.404 (0.198, 0.827)	0.013
Monthly family income					
750 Birr	41	8	1	1	
751–1500 Birr	26	10	0.242 (0.116, 0.502)	0.913 (0.18, 4.532)	0.01
>1500 Birr	57	76	0.310 (0.138, 0.694)	0.362 (0.14, 1.89)	0.021
ART adherence					
Poor	63	21	7.48 (3.26–11.77)	6.15 (3.88–9.69)	0.001*
Good	37	97	1	1	
WHO clinical stage of a child living with HIV/AIDS					
Stage I	59	72	1	1	
Stage II	15	8	2.30 (1.54–4.77)	2.10 (1.41–4.44)	0.12
Stage III	26	7	4.53 (2.57, 9.50)	3.50 (2.32–7.79)	0.06
Stage IV	24	7	4.18 (2.82, 6.021)	3.67 (1.52, 5.377)	0.08
Family size					
<4	55	69	1	1	
4–6	43	22	2.45 (3.26, 7.17)	1.81 (0.99, 2.98)	0.01
>6	26	3	10.61 (1.20, 16.28)	4.78 (0.49, 12.90)	0.037
Opportunistic infections					
Yes	27	10	2.34 (1.97–4.04)	2.01(1.88–2.98)	0.002*
No	97	84	1	1	
Co-trimoxazole prophylaxis					
No	111	56	5.8 (3.65–7.20)	4.66 (3.24–6.11)	0.001*
Yes	13	38	1	1	
Feeding frequency					
Twice	40	12	4.7 (2.88–7.46)	3.88 (1.94–5.14)	0.002*
Three times	57	44	1.82 (1.33–4.65)	1.52 (1.23–3.89)	0.04*
Four times	27	38	1	1	

AOR, adjusted odds ratio; ART, anti-retroviral therapy; COR, crude odds ratio.

## Discussion

The level of stunting among children living with HIV was 56.9% at the time this study was conducted. The high level of stunting in the study population is a cause for concern. This prevalence was comparable to previous studies, including Hyderabad, India^16^, Mozambique^17^, but it was lower than studies of Bobo-Dioulasso City, Burkina Faso^18^, Central India^19^, 2016’s Ethiopian Demographic And Health Survey (EDHS)^20^, Adama Hospital^21^, Northern Ethiopia^22^, Laquintinie Hospital, Douala, Cameroon^23^, and the West African Pediatric Cohort^24^.

On the other hand, our finding of the level of stunting was also higher than the rate of stunting [40%] among children reported from sub-Saharan Africa and 39% reported from South Asia^25^, El Salvador^26^, Lagos^27^, Harar^12^, Kenya^28^, Northern Ethiopia^29^, and Northwest Ethiopia’s multi-centre Study^30^. The likely reasons for the high burden of stunting among children living with HIV/AIDS in this study area might be study design, socio-economic, and study setting variations. The prevalence of stunting has large geographic disparities in Ethiopia, and the highest is in the Amhara region, where the current study was conducted^31^.

Poor ART adherence was found to be significantly associated with stunting. This is because treatment adherence is regarded as an important factor in achieving optimal outcomes across many disease states. In the treatment of HIV, poor adherence to treatment has the potential to impact outcomes on multiple levels^32^. Previous studies further supplement this. As per the study done in Uganda, the mean standardized weight-for-age z-score and weight-for-age percentiles both improved after the start of ART, with the z-score and weight-for-age percentile both considerably improving^33^, besides poor anti-retroviral medication adherence, it was found to be linked to severe low stature, according to a study in Botswana^34^.

Opportunistic infection was a significant predictor for stunting among children living with HIV/AIDS. To combat stunting in children effectively, opportunistic infections must be identified and treated early. This finding has been further cemented by previously published studies of a multi-centre study in Northwest Ethiopia^30^ and Zimbabwe^35^. Although co-trimoxazole prophylaxis was associated with stunting in this study, co-trimoxazole alters the gut microbiome and immune activation to lower systemic inflammation in HIV infection^36^; thereby, in the long term, it reduces mortality and morbidity from opportunistic infection due to low immunity^37^.

Low feeding frequency was associated with stunting. Feeding frequency ameliorates stunting among HIV/AIDS-infected children. Indeed, feeding frequency is vital for body physiology and in reducing malnutrition, including stunting^38^. To improve feeding frequency, concrete interventions should target children in poor households.

## Conclusion

In conclusion, the level of stunting among HIV-positive children was high. This might have its own impact on the morbidity and mortality of children living with HIV. Hence, the high prevalence of stunting in children living with HIV/AIDS in this study reflects the need for an expanded effort to address it intensively. Poor ART adherence, lack of co-trimoxazole prophylaxis, opportunistic infection, and low feeding frequency were significantly associated with stunting among HIV/AIDS-infected children. Interventions need to be targeted to improve ART adherence, early prophylaxis, the treatment of opportunistic infections, and feeding frequency.

## Limitations of the study

Due to the cross-sectional study design’s inherent difficulty in establishing a causal relationship between stunting and independent factors, one of the study’s main limitations was this. This work may significantly underestimate or overestimate the extent of stunting and other independent variables without follow-up observational data. Covariates such as the quantity of CD4 cells, the number of lymphocytes, the immunoglobulin status, the role of micronutrients, and the state of defecation were not considered.

## Ethical approval and consent to participate

All the methods were carried out in accordance with relevant guidelines and regulations. Ethical approval was obtained from an Institutional Review Board of Debre Markos University (Reference number: EH/880/15/14). A permission letter was obtained from the two hospitals. Written informed consent was obtained from the patient’s parents/legal guardian for publication and any accompanying images. A copy of the written consent is available for review by the Editor-in-Chief of this journal on request. The privacy of interviewees’ personal information was protected, and participants were made aware that they had the ability to decline or stop taking part in the study at any time.

## Consent to publish

Not applicable.

## Source of funding

No fund was obtained for this study.

## Author contribution

Z.D., Z.M., E.T., C.T., Z.A., A.A. and T.T. have been involved in the proposal development, and participated in statistical analysis. A.T., G.Y., L.K., K.A., B.A. and A.T. prepared the manuscript. E.T., Y.B. reviewed and approved the final manuscript.

## Conflicts of interest disclosure

The authors declare that they have no computing interest.

## Research registration unique identifying number (UIN)

Your unique identifying number is: researchregistry9358, and you will find your registration here: https://www.researchregistry.com/browse-theregistry#home/

## Guarantor

Eniyew Tegegne.

## Data availability statement

The dataset is accessible at the corresponding author upon request.

## Provenance and peer review

Not applicable.
